# Oncolytic Coxsackievirus B3 Strain PD-H Is Effective Against a Broad Spectrum of Pancreatic Cancer Cell Lines and Induces a Growth Delay in Pancreatic KPC Cell Tumors In Vivo

**DOI:** 10.3390/ijms252011224

**Published:** 2024-10-18

**Authors:** Anja Geisler, Babette Dieringer, Leslie Elsner, Robert Klopfleisch, Jens Kurreck, Henry Fechner

**Affiliations:** 1 Department of Applied Biochemistry, Institute of Biotechnology, Technische Universität Berlin, 10623 Berlin, Germanyhenry.fechner@tu-berlin.de (H.F.); 2 Institute of Veterinary Pathology, Freie Universität Berlin, 14163 Berlin, Germany

**Keywords:** pancreatic cancer, oncolytic virus, coxsackievirus, KPC cells, PD-H, CVA21

## Abstract

Pancreatic cancer is one of the deadliest cancers globally, with limited success from existing therapies, including chemotherapies and immunotherapies like checkpoint inhibitors for patients with advanced pancreatic ductal adenocarcinoma (PDAC). A promising new approach is the use of oncolytic viruses (OV), a form of immunotherapy that has been demonstrated clinical effectiveness in various cancers. Here we investigated the potential of the oncolytic coxsackievirus B3 strain (CVB3) PD-H as a new treatment for pancreatic cancer. In vitro, PD-H exhibited robust replication, as measured by plaque assays, and potent lytic activity, as assessed by XTT assays, in most pancreatic tumor cell lines, outperforming two other coxsackievirus strains tested, H3N-375/1TS and CVA21. Thus, H3N-375/1TS showed efficient replication and lytic efficiency in distinctly fewer tumor cell lines, while most tumor cells were resistant to CVA21. The oncolytic efficiency of the three OV largely correlated with mRNA expression levels of viral receptors and their ability to induce apoptosis, as measured by cleaved caspase 3/7 activity in the tumor cells. In a syngeneic mouse model with subcutaneous pancreatic tumors, intratumoral administration of PD-H significantly inhibited tumor growth but did not completely stop tumor progression. Importantly, no virus-related side effects were observed. Although pancreatic tumors respond to PD-H treatment, its therapeutic efficacy is limited. Combining PD-H with other treatments, such as those aiming at reducing the desmoplastic stroma which impedes viral infection and spread within the tumor, may enhance its efficacy.

## 1. Introduction

Pancreatic cancer currently has the lowest survival rate of all cancers [1]. Early-stage pancreatectomy remains the only effective treatment modality for the management of the disease and the only potential cure [2]. However, even with “curative” resection and concomitant chemotherapy, the 5-year survival rate for patients with PDAC is less than 25% [3]. Chemotherapy, primarily gemcitabine, has been used as first-line treatment for metastatic PDAC for nearly 25 years [4]. Various combination therapies, such as FOLFIRINOX (a combination of folic acid, oxaliplatin, fluorouracil and irinotecan) have shown better therapeutic results than gemcitabine [5]. Despite the efforts, metastatic PDAC has a very poor prognosis, with a median one-year survival rate of only 7% [6]. Immunotherapy with checkpoint inhibitors represents a new therapeutic approach, raising great hopes for the treatment of PDAC. However, clinical trials using programmed cell death-1 (PD-1), PD-ligand 1 (PD-L1), and cytotoxic T-lymphocyte-associated protein 4 (CTLA4) antagonists as monotherapies, as well as CAR-T-cell therapy, have not yet provided substantial benefits for PDAC patients [7,8]. The highly immunosuppressive tumor microenvironment (TME) of PDAC, along with its low expression of neoantigens, makes it a “cold” and less immunogenic tumor, contributing to the limited efficacy of these immunotherapies [8,9,10]. In addition, PDAC has an extremely dense stroma, which acts as a physical barrier to the delivery of anticancer drugs to the tumor cells [11].

OV represent an innovative and promising new approach to cancer treatment, as they have the potential to convert a “cold” tumor into a “hot” one. Their antitumor efficacy is based on two closely related mechanisms: virus-mediated killing of cancer cells leading to immunogenic cell death, and the activation of both innate and adaptive antitumor immune responses [12]. The uptake of OV into tumor cells, their replication within these cells, and virus-induced tumor cell lysis are crucial prerequisites for the induction of a strong antitumor immune response, thereby contributing to the therapeutic success of cancer treatment with OV [13,14]. During tumorigenesis, cancer cells acquire abnormalities in cellular metabolic pathways that enhance their susceptibility to OV. These include increased viral receptor expression [14,15,16], activation of the RAS/Raf1/MEK/ERK signaling pathway [17,18], or malfunction of the Janus kinase (JAK)/STAT [19] and type I interferon signalling pathways [20,21]. Due to the diverse interactions with these signalling pathways, cancer cells can exhibit varying levels of sensitivity to different OV [14,17,22]. Several OV have shown oncolytic activity in preclinical models of pancreatic cancer, including adenovirus, reovirus, vaccinia virus, vesicular stomatitis virus, herpes simplex virus, measles virus, parvovirus, influenza A virus, and Newcastle disease virus. Some of them, such as VCN-01, H101, LOAd703, VG161, Reolysin, and H-1PV have already reached phase II clinical trials, often in combination with chemo- or/and immunotherapy [23,24,25]. However, while OV therapy for pancreatic cancer demonstrates promising results in targeting cancer cells and stimulating antitumor immune responses, its ability to eliminate tumors and extend patient survival remains limited [24].

CVB3 is a new OV of the picornavirus family. It was first described in 2012 by Miyamoto et al. [17], who demonstrated strong growth inhibition of non–small cell lung tumors in a mouse xenograft model. Subsequent studies confirmed the antitumor activity of several oncolytic CVB3 strains in colorectal, breast, and endometrial cancer in immunodeficient and immunocompetent mouse cancer models [26,27,28]. Initial studies with oncolytic CVB3 for cancer treatment revealed that some strains could cause pancreatitis and myocarditis in vivo. However, this was prevented by equipping the viruses with microRNA (miR) target sites that are recognized by miRs highly expressed in the pancreas or heart but only minimally expressed or absent in tumor cells [26,29,30,31,32]. We have developed the oncolytic CVB3 strain PD-H, which has shown potent oncolytic activity in colorectal cancer in vivo [30]. Compared to other oncolytic CVB3 strains, PD-H exhibits a broader cancer cell tropism and induces fewer side effects [27,31]. This is strongly associated with its unique receptor tropism, as PD-H can infect cells by binding to N- and 6-*O*-sulfated heparan sulfates (HS) [27,33,34], in addition to utilizing the coxsackievirus and adenovirus receptor (CAR) for entry, which is also used by other oncolytic CVB3 strains. As a further OV with excellent oncolytic activity in colorectal cancer in vivo, we have developed H3N-375/1TS. This virus is derived from the CVB3 strain H3 [35], which is known to cause severe pancreatitis and myocarditis in mice. To mitigate these side effects, two target sites for the pancreas-specific miR-375 and two for the heart-specific miR-1 were incorporated into the 3′ UTR of the H3 genome, resulting in the modified virus variant H3N-375/1TS [30]. Treatment of mice with this virus demonstrated significant inhibition of tumor growth in a murine xenograft model of colorectal carcinomas, without any observed virus-induced side effects [30].

Here we investigated the oncolytic efficacy of PD-H in pancreatic cancer. Moreover, we compared its oncolytic activity with that of H3N-375/1TS and another oncolytic coxsackievirus, CVA21, which has shown oncolytic efficacy in several cancer models in vivo [36] and in clinical trials for treating malignant melanoma and non-muscle invasive bladder cancer [37,38]. We show that PD-H exhibits higher oncolytic efficiency in pancreatic cancer cells in vitro compared to both H3N-375/1TS and CVA21 and induces a growth delay in murine pancreatic KPC cell tumors in vivo.

## 2. Results

### 2.1. PD-H Exhibits Higher Lytic Activity and Stronger Replication in Pancreatic Cancer Cell Lines Compared to H3N-375/1TS and CVA21

**Figure 1:**
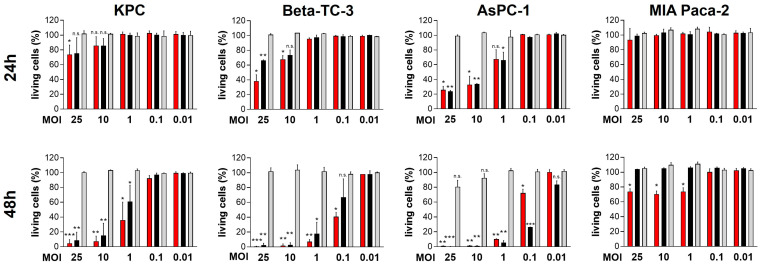

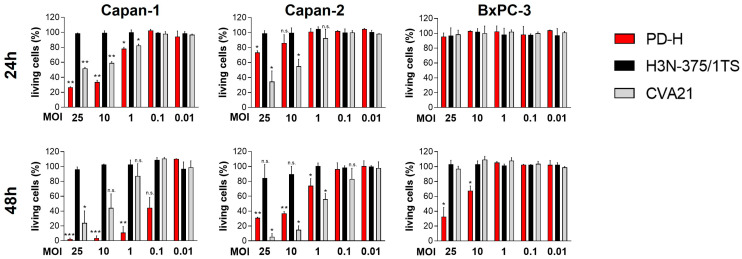
Oncolytic activity of PD-H, H3N-375/1TS, and CVA21 in pancreatic tumor cells. Cell viability: Pancreatic tumor cell lines (KPC, Beta-TC-3, AsPC-1, MIA Paca-2, Capan-1, Capan-2, and BxPC-3) were seeded in 96-well plates and infected with PD-H, H3N-375/1TS, or CVA21 at the indicated MOI. Cell viability was determined by XTT assay 24 and 48 h post-infection and is set relative to untreated cells (control). Data are shown as mean values ± SD from 2–3 independent experiments with three replicates each. Statistical significance of differences compared to control: * *p* < 0.05, ** *p* < 0.01 and *** *p* < 0.001; n.s., not significant.

We have been working with oncolytic CVB3 for several years and have demonstrated that the strains PD-H and H3N-375/1TS possess potent antitumor properties in the treatment of colorectal cancer in vivo [30,31]. However, PD-H and H3N-375/1TS differ in key characteristics, such as receptor tropism, which could ultimately influence their oncolytic activity. Since it was unclear how these differences would affect the oncolytic efficiency of the viruses in pancreatic cancer, we investigated both viruses in parallel. For the same reason, we also included CVA21 as another oncolytic coxsackievirus in our study. To determine the susceptibility of pancreatic cancer cell lines to PD-H, H3N-375/1TS and CVA21, we infected five human (AsPC-1, MIA Paca-2, Capan-1, Capan-2, BxPC-3) and two murine pancreatic (Beta-TC-3 and KPC) tumor cell lines with these viruses at MOI 0.01 to 25. The cell viability measured by an XTT assay 48 h after infection at MOI 1 provided a reliable assessment of whether a cell line is highly, moderately, or less sensitive to the viral infection, as these infection parameters are well-known and well-established for the investigated viruses. PD-H effectively lysed three of the seven cell lines tested (Beta-TC-3, AsPC-1, Capan-1), as indicated by a reduction in cell viability to less than 20% after 48 h and MOI 1. The cell lines KPC, Capan-2 and MIA Paca-2 showed moderate sensitivity with 40% to less than 80% cell viability. In contrast, BxPC-3 was resistant to PD-H at MOI 1 after 48 h. However, when the MOI was increased from 10 to 25, cell viability decreased from 70% to approximately 30% (Figure 1).

Using the same classification as above, two cell lines (Beta-TC-3, AsPC-1) were highly sensitive to H3N-375/1TS with a reduction in cell viability to less than 20% after 48 h. KPC cells showed moderate sensitivity to H3N-375/1TS with a remaining cell viability of 59%, while the other four cell lines (MIA Paca-2, Capan-1, Capan-2 and BxPC-3) were resistant.

**Figure 2:**
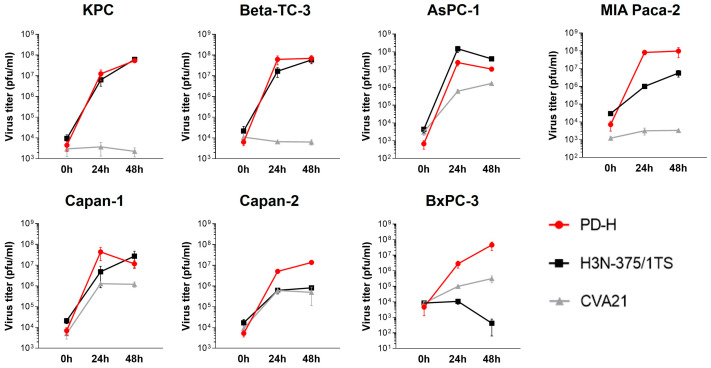
Virus growth curves kinetics of PD-H, H3N-375/1TS, and CVA21 in pancreatic tumor cells. The indicated pancreatic tumor cell lines were seeded in 96-well plates and infected with 1 MOI of PD-H, H3N-375/1TS, or CVA21. The virus was isolated at the indicated time points through three freeze/thaw cycles, and the virus titer was determined by plaque assay on HeLa cells. Data are shown as mean values ± SD from 2–3 independent experiments with two replicates each.

In contrast to PD-H and H3N-375/1TS, all pancreatic tumor cell lines were resistant to CVA21, except for Capan-2, which showed moderate sensitivity with a cell viability of 59% at MOI 1 after 48 h. In four of the seven cell lines (KPC, Beta-TC-3, MIA Paca-2, BxPC-3), no cell lysis was detectable, even at MOI 25. In two cell lines, increased cell lysis was observed, but only at a high MOI of 10 for Capan-1 or a very high MOI of 25 for AsPC-1. In addition to its ability to induce cytolysis, viral replication is a second important factor determining the oncolytic potential of an OV. To evaluate viral replication, we infected the pancreatic tumor cell lines with the three OV at MOI 1 and analyzed viral titers at 24 and 48 h post-infection. The viral titers measured 24 h post-infection were used to evaluate the replication efficiency of the viruses. At this time point, only a slight reduction in cell viability was observed for Capan-1 and AsPC-1, and no cell lysis was detected in the other cell lines (Figure 1). Thus, at this early stage, cell death had minimal impact on viral replication. We detected robust replication of PD-H across all pancreatic cancer cell lines, yielding high viral titers ranging from 10^6^ to 10^8^ pfu/mL. In comparison, H3N-375/1TS exhibited similar replication levels to PD-H in KPC, Beta-TC-3, and AsPC-1 cells. However, viral titers were approximately 8- to 9-fold lower in Capan-2 and Capan-1 and 81-fold lower in MIA Paca-2 cells, with no detectable replication in the BxPC-3 cell line. CVA21, on the other hand, did not replicate in KPC, Beta-TC-3, or MIA Paca-2 cells. Replication was observed in AsPC-1, Capan-1, Capan-2, and BxPC-3 cells, but the resulting viral titers were 8- to 41-fold lower than those achieved by PD-H (Figure 2).

In summary, these data clearly show that pancreatic cancer cell lines are overall highly and moderately sensitive to PD-H, moderately sensitive to H3N-375/1TS, but almost completely resistant to CVA21. However, virus replication only partially correlated with the lytic activity of the OV (Table 1).

### 2.2. Viral Receptors of PD-H, H3N-375/1TS and CVA21 Are Highly Variably Expressed in Pancreatic Cancer Cells

**Figure 3:**
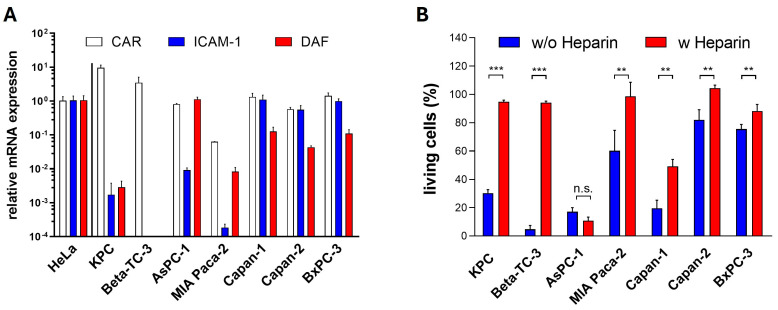
Viral receptor expression in pancreatic tumor cells. (**A**) Relative expression levels of CAR, ICAM-1, and DAF: The expression levels of CAR, ICAM-1, and DAF were determined by qRT-PCR. Each receptor’s expression level was normalized to the endogenous HPRT expression level and is set relative to the corresponding level in HeLa cells, which was set to 1. Data are shown as mean values ± SD from 2 independent samples with two replicates each. (**B**) Effect of heparin on PD-H infection in pancreatic tumor cell lines: PD-H at MOI 1 (MOI 10 for BxPC-3) was incubated with DMEM containing heparin (5000 µg/mL) or without heparin for 1 h before being applied to the cells. Cell viability was measured 48 h post-infection using the XTT assay. Data are shown as mean values ± SD from 3 independent experiments with three replicates each. Statistical significance as indicated: ** *p* < 0.01 and *** *p* < 0.001; n.s., not significant.

To elucidate mechanisms responsible for the differing sensitivity of pancreatic cancer cell lines to the investigated OV, we first examined the expression of viral receptors, as their expression is a decisive prerequisite for cellular infection. We assessed the expression of CAR, used by the CVB3 strains PD-H and H3N-375/1TS for cell entry, and ICAM-1 (intercellular adhesion molecule 1), the entry receptor for CVA21. We also measured the expression of DAF (decay accelerating factor), which functions as an attachment receptor for all three viruses. We also investigated the role of HS in PD-H’s ability to infect pancreatic cells, as PD-H is the only CVB3 that utilizes HS in addition to CAR for cell entry [27]. The expression of CAR, ICAM-1 and DAF in pancreatic tumor cell lines was quantified by real-time RT-PCR (qRT-PCR) and compared with their expression levels in HeLa cells, which are highly susceptible to all three viruses. Except for MIA Paca-2, which showed a 15-fold lower CAR expression, and KPC cells, which exhibited a 10-fold higher CAR expression than HeLa cells, the remaining five cell lines had CAR expression levels similar to those of HeLa cells. ICAM-1 expression was significantly more variable than CAR. High ICAM-1 levels comparable to HeLa cells were observed only in Capan-1, Capan-2, and BxPC-3 cells. In the other pancreatic cancer cell lines, ICAM-1 expression was 100- to over 5000-fold lower than in HeLa cells, and it was not detectable in Beta-TC-3 cells. DAF expression was also highly heterogeneous among the pancreatic tumor cell lines. Compared to HeLa cells, DAF expression was strong only in AsPC-1 cells. In contrast, DAF expression in Capan-1, Capan-2, and BxPC-3 was 8- to 23-fold lower, and in KPC and MIA Paca-2 cells, it was even 350- and 120-fold lower than in HeLa cells, respectively. DAF mRNA was not detected in Beta-TC-3 cells (Figure 3A).

To determine whether the uptake of PD-H in pancreatic cancer cell lines is mediated by HS, a heparin binding assay was performed. PD-H was incubated with the soluble HS-analogue heparin prior to infection, and cytotoxic activity was assessed using an XTT assay 48 h later. Blocking the interaction of PD-H with HS on the surface of pancreatic tumor cells led to a significant reduction of cell death in six of seven cell lines. Moreover, in five of these cell lines, cell death was almost completely prevented following pretreatment with heparin, highlighting the importance of HS for the infection of pancreatic cancer cell lines by PD-H (Figure 3B).

In summary, these data indicate that the expression of viral receptors largely correlates with the susceptibility of pancreatic cancer cell lines to PD-H, H3N-375/1TS and CVA21 (Table 1).

### 2.3. PD-H Induces Apoptosis in All Pancreatic Cancer Cell Lines, While H3N-375/1TS and CVA21 Induce Apoptosis in Only a Subset of Pancreatic Cancer Cell Lines

**Figure 4:**
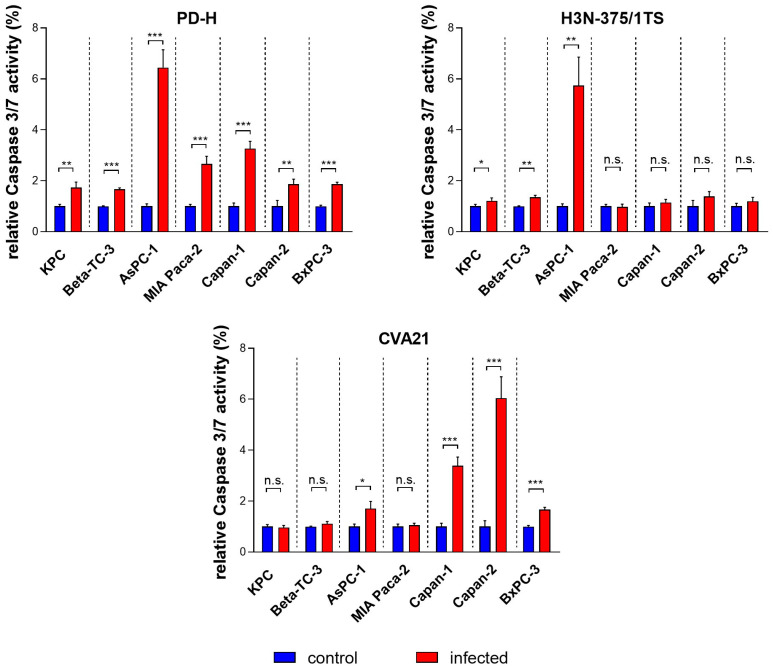
Relative cleaved caspase 3/7 activity in pancreatic tumor cells upon infection with PD-H, H3N-375/1TS, and CVA21. Pancreatic tumor cells were seeded in 96-well plates and infected with PD-H, H3N-375/1TS, or CVA21 at MOI 1. Cleaved caspase 3/7 activity was measured 24 h post-infection and normalized to the activity in untreated cells (control). Data are shown as mean values ± SD from 3 independent experiments with three replicates each. Statistical significance compared to control as indicated: * *p* < 0.05, ** *p* < 0.01 and *** *p* < 0.001; n.s., not significant.

The induction of apoptosis in infected tumor cells is a key mechanism by which OV exert their antitumor effects. This process not only facilitates the targeted destruction of tumor cells but also activates the immune system and supports viral replication and spread. To assess the potential of PD-H, H3N-375/1TS, and CVA21 to induce apoptosis, pancreatic tumor cell lines were infected with these viruses, and activated caspase 3/7 was measured using a cleaved caspase 3/7 assay 24 h post-infection. PD-H significantly induced apoptosis in all investigated cell lines, as indicated by an increase in cleaved caspase 3/7 activity. However, the extent of this increase varied among the cell lines: it was strong in AsPC-1 cells, moderate in MIA Paca-2 and Capan-1 cells, and low in KPC, Beta-TC-3, Capan-2, and BxPC-3 cells. In contrast, infection with H3N-375/1TS led to a significant increase in activated caspase 3/7 only in three of the seven pancreatic tumor cell lines: KPC, Beta-TC-3, and AsPC-1. Except for AsPC-1, the level of increase in these cell lines was lower than that observed with PD-H. Similarly, CVA21 treatment resulted in a significant increase in cleaved caspase 3/7 activity in only some of the pancreatic tumor cell lines with a strong increase observed in Capan-2 cells, a moderate increase in Capan-1, and a slight increase in AsPC-1 and BxPC-3 cells. No apoptosis induction was detected in the remaining three cell lines (Figure 4).

These data show that the three OV differ in their ability to induce apoptosis, both in terms of the range of affected cell lines and the strength of induction. Moreover, PD-H outperformed H3N-375/1TS and CVA21 in both aspects (Table 1).

### 2.4. PD-H Inhibits Growth of Subcutaneous Pancreatic KPC Tumors in a Syngeneic Mouse Model In Vivo

**Figure 5:**
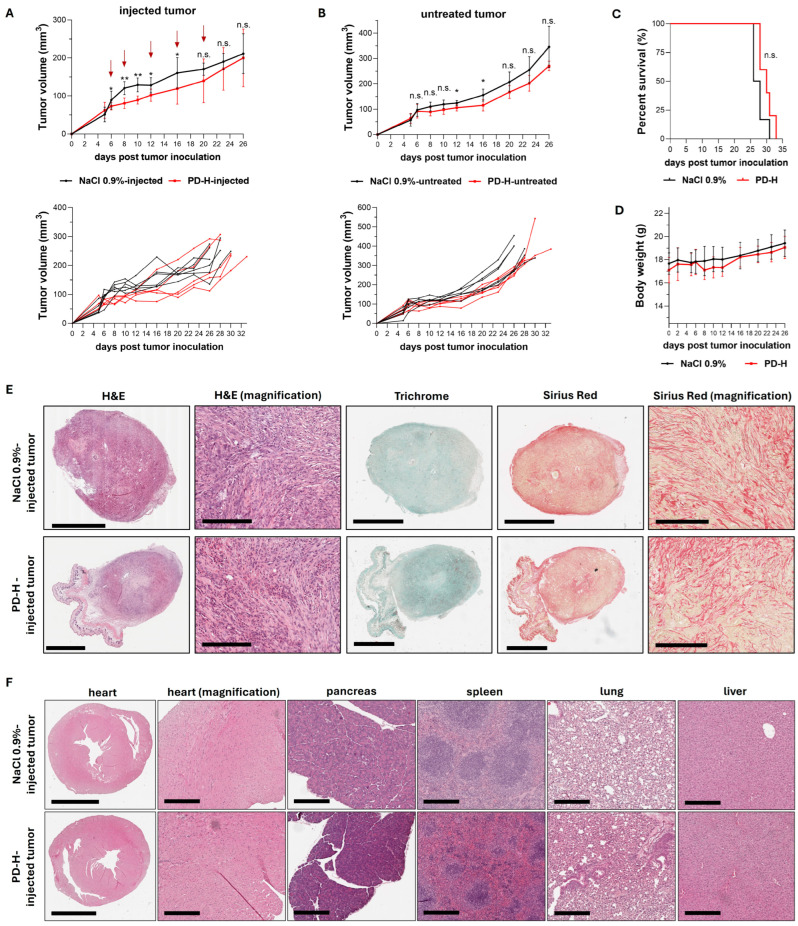
Oncolytic efficiency of PD-H in a syngeneic KPC tumor mouse model. Subcutaneous KPC tumors were established on both flanks of C57BL/6J mice. When the tumors reached a volume of 60–100 mm^3^, one of the tumors was injected with 3 × 10^6^ pfu of PD-H (n = 5) or 0.9% NaCl solution (n = 6), while the contralateral tumor remained untreated. The same dose of PD-H was administered on Days 2, 6, 10 and 14 post-initial injection. (**A**) Growth of the injected tumor: Shown are the mean values ± SD for each group (upper panel) and for each individual animal (lower panel). Statistical significance between PD-H-injected vs. NaCl 0.9%-injected as indicated: * *p* < 0.05, ** *p* < 0.01; n.s., not significant. Red arrows indicate the time points of PD-H/NaCl 0.9% injection. (**B**) Growth of the non-injected contralateral tumor: Shown are the mean values ± SD for each group (upper panel) and for each individual animal (lower panel). Statistical significance between untreated PD-H and untreated NaCl 0.9% tumors as indicated: * *p* < 0.05; n.s., not significant. (**C**) Kaplan–Meier survival curve; n.s., not significant. (**D**) Development of animal body weight. Shown are the mean values ± SD for each group. (**E**) Histological examination of the KPC tumor. Representative tumor slides stained with hematoxylin and eosin (H&E), Trichrome, and Sirius Red from a PD-H-injected tumor and a 0.9% NaCl-treated tumor on Day 28 post-tumor inoculation. Scale bars = 2 mm. Magnification scale bars = 200 µm. (**F**) Histological examination of murine tissues. Representative slides of murine organs (heart, pancreas, spleen, lung, liver) stained with H&E are shown on Day 26 (0.9% NaCl-treated) and Day 28 (PD-H-treated) after tumor inoculation. Scale bars = 2 mm (heart) and 300 µm (magnification heart, pancreas, spleen, lung, liver).

Encouraged by our findings demonstrating the broad and strong oncolytic efficacy of PD-H in pancreatic cancer cell lines in vitro, we next investigated the antitumor efficiency of PD-H in vivo. We established a syngeneic subcutaneous pancreatic tumor model by injecting murine pancreatic KPC tumor cells into both flanks of C57BL/6J mice. The KPC cell line was generated by culturing pancreatic tumor tissue from the genetically modified C57BL/6J-KPC mouse, a well-validated and clinically relevant model of PDAC. It develops many key features observed in human PDAC such as pancreatic intraepithelial neoplasia, metastasis to the liver and lungs, and the exclusion of effector T cells [39]. When the subcutaneous KPC cell tumors reached a volume of approximately 60–100 mm^3^, one tumor was injected with 3 × 10^6^ pfu of PD-H. Intratumoral (i.t) injections were repeated on Days 2, 6, 10 and 14 post-initial injection using the same PD-H dose. Control mice received i.t. injections of 0.9% NaCl solution at the same time points. Compared to the control group, PD-H treatment significantly inhibited tumor growth in both the PD-H-injected and the non-injected contralateral tumor. However, the inhibition of tumor growth was moderate for both tumors (Figure 5A,B). The survival rate of PD-H-treated animals tended to be higher than that of the control group, but the difference was not statistically significant (Figure 5C).

Mice were primarily sacrificed due to the development of necrosis and ulcers in the skin over the tumors, rather than due to tumor size or general health deterioration. At the time of sacrifice (Days 26 to 33 post-tumor inoculation), we tested the PD-H-injected tumors for replicating virus using plaque titration on HeLa cells, but no virus was detected, indicating that PD-H had been completely cleared by the host’s immune system. No significant changes in body weight were observed between treated and control animals (Figure 5D). Histological examinations of the tumors showed that the tumor cells were present within encapsulated areas interspersed with strong fibrotic tissue strands, with no differences in tumor architecture between PD-H-injected and control animals (Figure 5E). To confirm the safety of PD-H treatment, we histologically examined murine organs, including the pancreas and heart, which are the most susceptible organs to CVB3. No pathological alterations were found (Figure 5F).

In summary, these data confirm the safety and oncolytic efficacy of PD-H in pancreatic cancer in vivo. However, the antitumor efficiency of PD-H was relatively low.

## 3. Discussion

PDAC is one of the most challenging tumor diseases to treat, with nearly all patients diagnosed with PDAC dying within five years. Therefore, there is an urgent need to develop new antitumor agents for affected patients. Immunotherapy has become a promising approach in cancer treatment, offering new hope, particularly when conventional treatments have failed. Among these, OV represent a unique class of antitumor agents that have already shown efficacy in clinical cancer treatments [40].

In this study, we investigated PD-H, an oncolytic CVB3 previously shown to have potent anticancer efficacy in colorectal carcinomas [31], for its oncolytic activity in pancreatic carcinomas. In vitro studies demonstrated that PD-H exhibited robust replication and cytolytic activity in pancreatic cancer cell lines, significantly outperforming the other two oncolytic coxsackieviruses tested, H3N-375/1TS and CVA21. Moreover, intratumoral injection of PD-H led to significant growth inhibition of murine pancreatic KPC tumors in a subcutaneous syngeneic carcinoma mouse model in vivo.

Our primary rationale for investigating PD-H and the two other oncolytic coxsackieviruses for their oncolytic activity in pancreatic cancer is based on the fact that these OV are among the smallest viruses, with a size of approximately 30 nm [41]. We hypothesized that their small size would enable them to more effectively penetrate the dense stroma and physical barriers of pancreatic tumors, a challenge that has limited the efficacy of larger viruses like adenoviruses and herpesviruses in clinical trials [42]. Additionally, coxsackieviruses have one of the shortest viral replication cycles, producing large quantities of new viral particles within 6–8 h post-infection [43]. This rapid replication should facilitate their rapid spread from infected to uninfected tumor cells, potentially improving therapeutic outcomes in pancreatic carcinoma treatment.

Efficient infection of tumor cells is a critical factor for strong antitumor efficacy and is primarily dependent on the expression of viral receptors. PD-H exploits both HS and CAR to enter tumor cells. Our studies confirm that while PD-H primarily utilizes HS for infecting pancreatic cancer cells, it can also use CAR, particularly in cell lines where HS-mediated infection is not supported, and CAR is expressed at high levels. This dual-receptor utilization is a decisive advantage of the virus, as it determines its broad tropism to different pancreatic cancer cells. The significance of this versatility becomes even clearer when considering the susceptibility of pancreatic cancer cell lines to H3N-375/1TS and CVA21. CVA21 exhibited low oncolytic efficacy in pancreatic cancer cells, likely due to a lack of or low expression of its receptor, ICAM-1. The oncolytic efficiency of H3N-375/1TS was higher than that of CVA21 but did not reach the efficiency of PD-H. This could be partly due to the fact that H3N-375/1TS uses CAR and not HS for cell entry. A notable exception was the BxPC-3 cell line, which exhibited strong resistance to infection by both CVA21 and H3N-375/1TS, despite expressing high levels of the relevant viral receptors. This discrepancy may arise from a lack of viral receptors on the cell surface, as the mRNA expression of CAR and ICAM-1 used in this study does not necessarily reflect the presence of sufficient functional receptor proteins on the tumor cell surface. However, it cannot be ruled out that other factors, such as intracellular inhibition of viral replication, contribute to the resistance of the cell line to CVA21 and H3N-375/1TS infections.

The ability of a virus to induce apoptosis is another factor that influences the oncolytic efficiency of OV. While treatment with all three coxsackieviruses resulted in an increase in apoptosis, PD-H was the only virus that consistently induced apoptosis in all pancreatic cancer cells. In contrast, treatment with CVA21 and H3N-375/1TS induced apoptosis in only about half of the cell lines examined. It is also important to note that CVB3 can trigger other forms of cell death in addition to apoptosis. One of these is pyroptosis, which occurs through the activation of caspase 3 via the cleavage of gasdermin E. This type of cell death was detected in colon cancer cell lines [44]. Whether CVB3 induces pyroptosis in pancreatic cancer cells is currently unknown.

H3N-375/1TS contains target sites for the heart-specific miR-1 and pancreas-specific miR-375 within its genome, which leads to inhibition of viral replication in both organs [30]. However, if these miRs are also expressed in pancreatic tumor cells, it is possible that viral replication, and thus oncolytic activity, could be diminished. Nonetheless, both miRs were found in very low quantities in the pancreatic tumor cell lines, meaning the reduced replication of H3N-375/1TS compared to PD-H cannot be attributed to the miR-TS. An exception may be the Capan-1 cell line, where miR-375 is expressed at comparatively high levels (Supplementary Figure S1). In this case, it is likely that the expression of miR-375 contributed to the inhibition of H3N-375/1TS replication, leading to reduced viral replication in this cell line.

Treating pancreatic cancer in animal model studies using OV has proven to be extremely challenging. The effectiveness of the therapy is influenced by various factors, including the cell line used to generate the tumor, the OV strain, the administered OV dose, the genetic background of the animals, and the route of virus administration [45,46]. To investigate the antitumoral efficiency of PD-H, we established a subcutaneous pancreatic tumor model using murine KPC cells. The i.t. injection of PD-H into subcutaneous KPC tumors led to a significant inhibition of tumor growth. Importantly, the antitumor effect was observed not only in the injected tumor but also in the contralateral, non-injected tumor, demonstrating systemic efficacy of the local PD-H treatment. However, it remains unclear whether this effect results from direct virus-induced cancer cell lysis or from virus-trigged anticancer immune response. Both mechanisms likely contribute, as demonstrated by our previous studies in colorectal cancer, which showed that PD-H can spread from its initial site of replication in the injected tumor to a distant, uninfected tumor [27,31]. Despite this systemic effect, the overall antitumor effect was moderate. There was no tumor regression or no halt of tumor progression, and animal survival was not improved. Based on the development of the KPC tumor after treatment, our data show that the initial PD-H injection was primarily responsible for the observed growth inhibition, with subsequent injections failing to enhance therapeutic outcome as expected. Typical of pancreatic cancer, KPC tumors have an extremely dense fibrotic stroma that penetrates and surrounds the tumor, accounting for up to 80% of the tumor mass [45]. This stroma not only promotes pancreatic tumor cell proliferation but also acts as a significant physical barrier to chemo-, radio-, and immunotherapies [47,48,49]. Its importance in our approach becomes evident, as we observed the transition of KPC tumors from being soft and injectable at the first PD-H injection to becoming increasingly hard and difficult to infect in subsequent injections. Regarding our initial hypothesis, even small viruses like PD-H, which efficiently infect pancreatic tumor cells, appear unable to overcome these barriers within pancreatic tumors.

Based on the data presented here, as well as preclinical and clinical investigations, it can be concluded that reducing fibrotic tissue is a crucial prerequisite to improve the efficacy of OV as a treatment for pancreatic cancer. Therefore, various promising approaches are being explored, such as the inhibition of the focal adhesion kinase, which is considered a key factor in the fibrotic and immunosuppressive TME [50]. Another approach targets hyaluronic acid, a major component of the dense extracellular matrix in pancreatic tumors, using hyaluronidase [45,51]. The reduction of stromal density could promote the penetration of OV into the tumor tissue, and it is likely that small OV as PD-H would benefit more from this strategy compared to larger viruses.

In summary, we have shown that the OV PD-H efficiently infects and kills pancreatic cancer cells in vitro and inhibits the growth of pancreatic KPC cell tumors in vivo. However, the therapeutic efficiency of the OV was only moderate. Reducing fibrotic tissue within the tumor appears essential to improve the efficiency of PD-H in treating pancreatic cancer. Ongoing studies aim to verify this.

## 4. Materials and Methods

### 4.1. Cell Culture

HEK293 (human embryonic kidney) cells were cultured in high glucose Dulbecco’s modified Eagle’s medium (DMEM; Biowest, Darmstadt, Germany) supplemented with 10% fetal calf serum (FCS; c.c. pro, Oberdorla, Germany), 1% L-glutamine (Sigma-Aldrich, Merck, Darmstadt, Germany), 1% sodium pyruvate (Sigma-Aldrich), and 1% each of penicillin and streptomycin (P/S; AppliChem, Darmstadt, Germany). HeLa cells were cultured in Minimum Essential Medium (MEM) (Gibco, Thermo Fisher Scientific Inc., Waltham, MA, USA) supplemented with 5% FCS, 0.02 M 2-[4-(2-hydroxyethyl)piperazin-1-yl]ethanesulfonic acid (HEPES; Sigma-Aldrich), 1% non-essential amino acids (NEAA) and 1% P/S. The KPC, Beta-TC-3 (murine pancreatic insulinoma beta-tumor), and MIA Paca-2 cell lines were cultured in DMEM high glucose with 10% FCS, 1% P/S and 1% L-glutamine. The AsPC-1, Capan-1, Capan-2 and BxPC-3 cell lines were grown in RPMI 1640 (c.c.pro) supplemented with 10% FCS, 1% P/S, and 1% L-glutamine.

### 4.2. Viruses

CVA21 (strain Kuykendall) was purchased from ATCC (Manassas, VA, USA) and amplified on HeLa cells with 0.3 MOI for 48 h. The oncolytic viruses CVB3 PD-H and H3N-375/1TS were produced as described previously [30,31]. Briefly, to generate PD-H, the plasmid pJET-CVB3-PD-H containing the cDNA of the CVB3 strain PD-H was linearized with *Pme*I, and purified plasmid DNA was reverse transcribed using the in vitro T7 Transcription Kit (Roboklon GmbH, Berlin, Germany). The purified RNA was then transfected in CHO-K1 cells using PEImax (Polysciences Europe GmbH, Hirschberg an der Bergstraße, Germany). PD-H was further amplified on CHO-K1 cells at MOI 0.3 for 48 h. H3N-375/1TS, which contains two copies each of the miR-375TS and miR-1TS in the backbone of the H3 strain of CVB3, was generated by transfection of the plasmid pMKS1-H3N-375/1TS into HEK293 cells using PEImax for 48 h. H3N-375/1TS was further amplified on HeLa cells with 5 MOI for 24 h. For virus release, infected cells were subjected to three freeze and thaw cycles, and the cell debris was removed by centrifugation. The supernatant was analyzed by plaque assay. To produce sufficient virus for in vivo experiments, PD-H was further amplified by infection of CHO-K1 cells, followed by virus release and concentration/purification via 30% sucrose gradient centrifugation as described previously [52]. The titers of all three viruses were determined by plaque assay on HeLa cells.

### 4.3. Virus Plaque Assay

Plaque assays were carried out on HeLa cells as described previously [53].

### 4.4. Cell Viability Assay

Cell viability was measured using the Cell Proliferation Kit (XTT; Promega GmbH, Walldorf, Germany) according to the instructions of the manufacturer. Cells were seeded in 96-well plates to reach approximately 80% confluence the following day. The cells were then infected with viruses at concentrations of 0.01 to 25 MOI at 37 °C for 1 h in serum-free medium. Cells that were not infected (control) were only incubated with serum-free medium. After the incubation period, the medium was replaced with fresh cell culture medium. Absorbance levels were measured 24, 48, and 72 h post-infection using the TriStar2 LB 942 Modular Multimode Microplate Reader (Berthold Technologies, Bad Wildbad, Germany). As a control for cell death, cells were treated with 50 µL 5% Triton X-100 solution (Carl Roth GmbH + Co. KG, Karlsruhe, Germany).

### 4.5. Virus Growth Curves

Cells were seeded into 96-well plates to reach approximately 80% confluence the following day. For infection, the medium was replaced by serum-free medium containing 1 MOI of PD-H, H3N-375/1TS, or CVA21. After incubating for 1 h at 37 °C, the virus suspension was replaced by fresh cell culture medium. Cells were immediately frozen (0 h) or at 24 and 48 h post-infection, followed by lysis through three freeze-thaw cycles. The cell debris was discarded by centrifugation, and the virus titers from the supernatant were analyzed by plaque assay.

### 4.6. Quantification of CAR, DAF, ICAM-1, and miR Expression

Total RNA was isolated from murine pancreas and heart tissue, pancreatic tumor cell lines, and HeLa cells using the TRIzol^®^ Reagent (Thermo Fisher Scientific Inc.). For quantification of receptor mRNA, 2 mg of RNA was treated with 2 U DNaseI (New England Biolabs, Frankfurt am Main, Germany) for 1–2 h at 37 °C. The High-Capacity cDNA Reverse Transcription Kit (Thermo Fisher Scientific Inc.) was used to reverse transcribe 500–1000 ng DNaseI-treated RNA. Expression levels of CAR (Mm00438355_m1, Hs00154661_m1), ICAM-1 (Mm00516023_m1, Hs00164932_m1), and DAF (Mm00438377_m1, Hs00892618_m1) were quantified using the TaqMan Gene expression Master Mix and TaqMan Gene Expression Assays (Thermo Fisher Scientific Inc.) according to the manufacturer’s instructions. HPRT1 (Mm01324427_m1, Hs02800695_m1) was used for normalization. For quantification of miR-1 (assay ID: 002222) and miR-375 (assay ID: 000564), 50 ng of total RNA was reverse-transcribed and amplified using the TaqMan MicroRNA Assays (Thermo Fisher Scientific Inc.) according to the manufacturer’s instruction, with U6 snRNA (assay ID: 001973) as the normalization control. qPCR reactions were carried out in duplicate in a C1000 Thermal Cycler and CFX96 real-time system (Bio-Rad, Feldkirchen, Germany). Data were analyzed using the DDCT calculation method.

### 4.7. Heparin Binding Assay

Heparin sodium salt from porcine intestinal mucosal (Sigma-Aldrich) was dissolved in DMEM to create a stock concentration of 10 mg/mL and stored at 4 °C. Cells were seeded into 96-well plates to reach approximately 80% confluence the next day. PD-H (MOI 1; BxPC-3 MOI 10) was incubated with DMEM containing heparin (5000 µg/mL) or without heparin for 1 h at 37 °C and then applied to the cells for 1 h at 37 °C. After a 30 min incubation at 37 °C, the virus suspension was replaced by fresh cell culture medium. Plates were incubated for 48 h and cell viability was determined using the XTT assay.

### 4.8. Determination of Caspase 3/7 Activities

Cleaved caspase 3/7 activity was measured using Caspase Glo^®^ 3/7 Assay Systems (Promega GmbH) according to the manufacturer’s instructions. Briefly, cells were seeded in 96-well plates and, upon reaching approximately 80% confluence the next day, were infected with 1 MOI of PD-H, H3N-375/1TS, or CVA21. Cells that were not infected (control) were only incubated with serum-free medium. After 24 h, luminescence was measured using the TriStar2 LB 942 Modular Multimode Microplate Reader (Berthold Technologies).

### 4.9. Histopathological Analysis

Tissues were fixed in 4% paraformaldehyde (Carl Roth GmbH + Co. KG) and embedded in paraffin. After cutting 5 μm thick tissue sections, they were stained with H&E. This staining allowed for the assessment of inflammation, tissue destruction due to pathology, and the basic structure and arrangement of cells within the sections. Trichrome and Sirius Red staining were performed to evaluate connective tissue and collagen expression.

### 4.10. Syngeneic Subcutaneous KPC Cancer Mouse Model

The animal experiments were performed in accordance with the principles of laboratory animal care and all German laws regarding animal protection and approved by the responsible local authorities (State Office of Health and Social Affairs, Berlin, Germany, reference number G 0048/18). For generation of KPC tumors, 6-week-old female C57BL/6J mice (Charles River, Sulzfeld, Germany) were injected with 5 × 10^6^ KPC cells (Ximbio, London, UK) subcutaneously into the right and left flank. When tumors reached a volume of 60–100 mm^3^, the right tumor was injected with 3 × 10^6^ pfu PD-H in a total volume of 30 µL. Control animals received 30 µL of 0.9% NaCl solution instead. In both the PD-H-treated and the control group, the contralateral tumor remained untreated. Tumor volume was measured using a caliper.

### 4.11. Statistical Analysis

Statistical analyses were performed using GraphPad Prism 8.4.3 (GraphPad Software, Boston, MA, USA). Statistical significance for in vitro data was determined by an unpaired Student’s *t*-test, while statistical significance for in vivo data was assessed using the Mann–Whitney U-test. Survival curves (Kaplan–Meier) were compared using the Log-Rank (Mantel-Cox) test. A *p*-value of <0.05 was considered statistically significant.

## Figures and Tables

**Table 1:** Overview of the susceptibility of pancreatic cancer cell lines to PD-H, H3N-375/1TS, and CVA21.

***Pancreatic Cancer Cell Line***	***Cell Death***			***Viral Replication***			***Receptor mRNA Expression***					***Apoptosis Induction***		
	***++++***	***>80% lysis***		***++++***	***up to 10^8^ pfu/ml***		***++++***	***~100–1000% to control***		***PD-H sensitive to heparin***		***+++***	***strong more than 5-fold***	
	***+++***	***>60% lysis***		***+++***	***up to 10^7^ pfu/ml***		***+++***	***~100% to control***		***++***	***strong***	***++***	***middle up to 5-fold***	
	***++***	***>40% lysis***		***++***	***up to 10^6^ pfu/ml***		***++***	***~10–100% to control***		***+***	***visible***	***+***	***low up to 2-fold***	
	***+***	***>20% lysis***		***+***	***up to 10^5^ pfu/ml***		***+***	***~1–10% to control***		***-***	***not sensitive***	***-***	***no significant induction***	
	***-***	***no lysis***		***-***	***no replication***		***-***	***below 1% to control***						
	**PD-H**	**H3N-375/1TS**	**CVA21**	**PD-H**	**H3N-375/1TS**	**CVA21**	**CAR**	**DAF**	**ICAM-1**			**PD-H**	**H3N-375/1TS**	**CVA21**
KPC	+++	++	-	++++	+++	-	++++	-	-	++		+	+	-
Beta-TC-3	++++	++++	-	++++	++++	-	++++	-	-	++		+	+	-
AsPC-1	++++	++++	-	++++	++++	++	+++	+++	-	-		+++	+++	+
MIA Paca-2	+	-	-	++++	+++	-	+	-	-	++		++	-	-
Capan-1	++++	-	-	++++	+++	++	+++	++	+++	+		++	-	++
Capan-2	+	-	++	+++	++	++	++	+	++	+		+	-	+++
BxPC-3	-	-	-	+++	-	+	+++	++	+++	+		+	-	+

Assessment according to the following criteria: cell death (MOI 1 for 48 h), viral replication (MOI 1 for 24 h), heparin-induced inhibition of PD-H (MOI 1 for 48 h, BxPC-3 at MOI 10), and apoptosis induction (MOI 1 for 24 h).

## Data Availability

Data will be supplied following reasonable requests.

## Supplementary Materials

The following supporting information can be downloaded at: https://www.mdpi.com/article/10.3390/ijms252011224/s1.
