# Supplementary material for: Oncolytic Coxsackievirus B3 Strain PD-H Is Effective Against a Broad Spectrum of Pancreatic Cancer Cell Lines and Induces a Growth Delay in Pancreatic KPC Cell Tumors In Vivo
Source: Int J Mol Sci. 2024 Oct 18;25(20):11224. doi: 10.3390/ijms252011224 (PMC11508574; doi:10.3390/ijms252011224)
Supplement: Supplementary file 1 [file ijms-25-11224-s001.zip › ijms-3228443-supplementary.pdf]

Supplementary Data

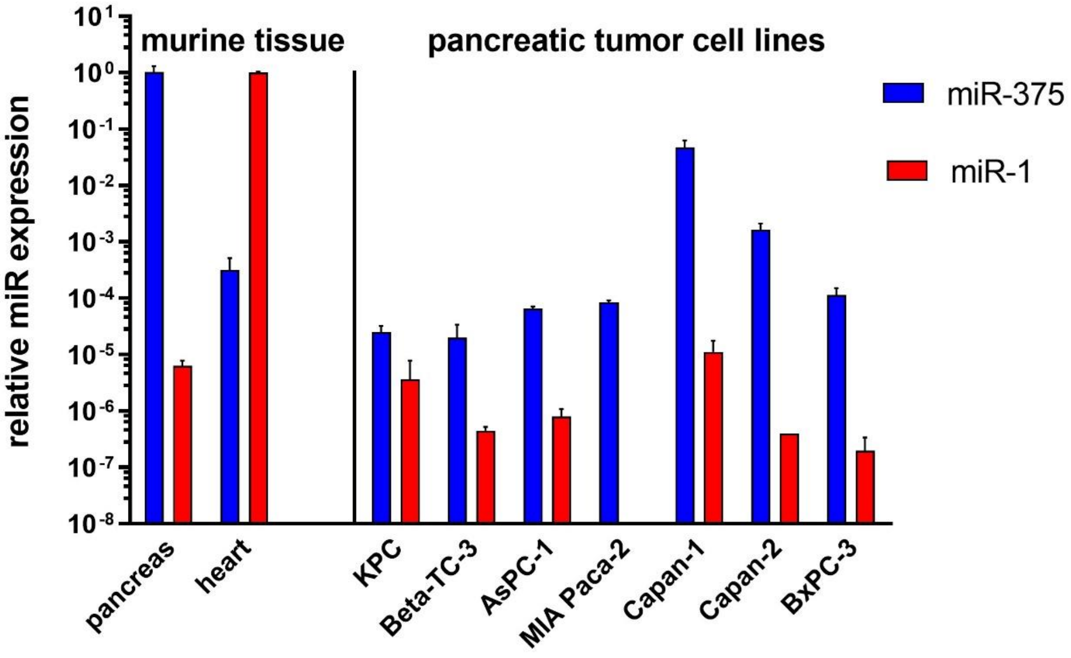

Figure S1: Expression of pancreas-specific expressed miR-375 and heart-specific expressed miR-1 in murine heart and pancreas tissue and in pancreatic tumor cell lines.

Total RNA was isolated from murine heart and pancreas and from seven different pancreatic tumor cell lines. Expression of miR-375 and miR-1 was measured by qRT-PCR and normalized to U6 snRNA expression. Data are shown as mean values ± SD from 2 independent samples with each two replicates.
